# Association of Neighborhood Resources and Race and Ethnicity With Readmissions for Diabetic Ketoacidosis at US Children’s Hospitals

**DOI:** 10.1001/jamanetworkopen.2022.10456

**Published:** 2022-05-05

**Authors:** Kelly R. Bergmann, Amanda Nickel, Matt Hall, Gretchen Cutler, M. Jennifer Abuzzahab, Brianna Bretscher, Shea Lammers, Dave Watson, Gabrielle Z. Hester

**Affiliations:** 1 Department of Pediatric Emergency Medicine, Children’s Minnesota, Minneapolis; 2 Department of Research and Sponsored Programs, Children’s Minnesota, Minneapolis; 3 Department of Analytics, Children’s Hospital Association, Overland Park, Kansas; 4 Department of Endocrinology, McNeely Diabetes Center, Children’s Minnesota, St Paul; 5 Department of Value and Clinical Excellence, Children’s Minnesota, Minneapolis

## Abstract

**Importance:**

The Child Opportunity Index 2.0 (COI) assesses neighborhood resources and conditions that influence health. It is unclear whether the COI scores are associated with health outcomes by race and ethnicity among children with type 1 diabetes (T1D).

**Objective:**

To determine whether COI categories are associated with diabetes-related outcomes by race and ethnicity, including readmissions for diabetic ketoacidosis (DKA) and co-occurring acute kidney injury (AKI) or cerebral edema (CE).

**Design, Setting, and Participants:**

This cross-sectional study included children discharged with a primary diagnosis of T1D with DKA between January 1, 2009, and December 31, 2018. Merged data were obtained from the Pediatric Health Information System and COI. Participants included children and adolescents younger than 21 years with an encounter for DKA. Data were analyzed from April 29, 2021, to January 5, 2022.

**Exposures:**

Neighborhood opportunity, measured with the COI as an ordered, categorical score (where a higher score indicates more opportunity), and race and ethnicity.

**Main Outcomes and Measures:**

The primary outcome was readmission for DKA within 30 and 365 days from an index visit. Secondary outcomes included the proportion of encounters with AKI or CE. Mixed-effects logistic regression was used to generate probabilities of readmission, AKI, and CE for each quintile of COI category by race and ethnicity.

**Results:**

A total of 72 726 patient encounters were identified, including 38 924 (53.5%) for girls; the median patient age was 13 (IQR, 9-15) years. In terms of race and ethnicity, 600 (0.8%) of the encounters occurred in Asian patients, 9969 (13.7%) occurred in Hispanic patients, 16 876 (23.2%) occurred in non-Hispanic Black (hereinafter Black) patients, 40 129 (55.2%) occurred in non-Hispanic White (hereinafter White) patients, and 5152 (7.1%) occurred in patients of other race or ethnicity. The probability of readmission within 365 days was significantly higher among Black children with a very low COI category compared with Hispanic children (risk difference, 7.8 [95% CI, 6.0-9.6] percentage points) and White children (risk difference, 7.5 [95% CI, 5.9-9.1] percentage points) at the same COI category. Similar differences were seen for children with very high COI scores and across racial groups. The COI category was not associated with AKI or CE. However, race and ethnicity constituted a significant factor associated with AKI across all COI categories. The probability of AKI was 6.8% among Black children compared with 4.2% among Hispanic children (risk difference, 2.5 [95% CI, 1.7-3.3] percentage points) and 4.8% among White children (risk difference, 2.0 [95% CI, 1.3-2.6] percentage points).

**Conclusions and Relevance:**

These results suggest that Black children with T1D experience disparities in health outcomes compared with other racial and ethnic groups with similar COI categories. Measures to prevent readmissions for DKA should include interventions that target racial disparities and community factors.

## Key Points

**Question:**

Is neighborhood opportunity differentially associated with health outcomes by race and ethnicity among US children with diabetic ketoacidosis?

**Findings:**

In this cross-sectional study including 72 726 pediatric encounters for diabetic ketoacidosis, the probability of readmission within 365 days was significantly higher among non-Hispanic Black children compared with Hispanic children and non-Hispanic White children at the same level of opportunity. Within racial and ethnic groups, children with very low opportunity had significantly greater probability of readmission compared with those with very high opportunity.

**Meaning:**

These findings suggest that despite having similar measures of neighborhood opportunity, non-Hispanic Black children with type 1 diabetes experience disparities in health outcomes compared with children in other racial and ethnic groups.

## Teaser

This cross-sectional study assesses whether neighborhood opportunity is associated with type 1 diabetes–related outcomes by race and ethnicity, including readmissions for diabetic ketoacidosis and co-occurring acute kidney injury and cerebral edema.

## Introduction

Diabetic ketoacidosis (DKA) is the leading cause of hospitalization, morbidity, and mortality in children with type 1 diabetes (T1D).^1,2^ Previous studies have identified racial and ethnic disparities among children with T1D, particularly regarding risk of DKA. A study from the Centers for Disease Control and Prevention^3^ found that non-Hispanic Black (hereinafter Black) children had a nearly 2-fold increased risk of diabetes-related mortality, which included deaths due to DKA, compared with non-Hispanic White (hereinafter White) children. Further, White children are less likely to require hospitalization for DKA compared with children of other races and ethnicities.^2,4^ Although socioeconomic factors—including lack of health insurance or public insurance,^2,4,5,6,7,8,9,10,11,12^ lower income or living in areas of poverty,^2,4,9,10,12^ and lower parental educational achievement^10^—have been associated with higher risk and severity of DKA, other community attributes may influence health outcomes.

The Child Opportunity Index 2.0 (COI) was developed in 2020 and is a novel instrument that provides a comprehensive assessment of neighborhood attributes that may elucidate structural inequities and provide a more robust and practical assessment of social factors that influence health outcomes.^13^ Studies have shown that greater neighborhood opportunity is associated with fewer acute care visits^14^ and hospitalizations for ambulatory care conditions.^15,16^ It is unclear whether neighborhood opportunity influences diabetes-related outcomes. To address this gap in knowledge, we evaluated whether COI categorical scores are associated with diabetes-related outcomes by race and ethnicity with regard to (1) readmissions for DKA and (2) complications of DKA, including acute kidney injury (AKI) and cerebral edema (CE).

## Methods

### Study Design, Participants, and Setting

We conducted a cross-sectional study of children and adolescents younger than 21 years who were discharged from inpatient, observation, or emergency department care with a primary diagnosis of T1D with ketoacidosis (*International Classification of Diseases, Ninth Revision* [*ICD-9*], codes 250.11 and 250.13 or *International Statistical Classification of Diseases and Related Health Problems, Tenth Revision* [*ICD-10*], codes E10.10 and E10.11) from January 1, 2009, to December 31, 2019. We excluded encounters from 2019 because these were used to determine readmission for 2018, duplicate encounters (ie, 2 encounters with the same admission date), and encounters with missing COI categorical scores or demographic information. This study followed the Strengthening the Reporting of Observational Studies in Epidemiology (STROBE) reporting guideline and was deemed exempt from review and the need for informed consent by the institutional review board of Childrens’ Minnesota.

### Data Sources

Clinical data were obtained from the Pediatric Health Information System (PHIS), which is managed by the Children’s Hospital Association, Lenexa, Kansas, and includes data from the largest US children’s hospitals. Data quality and reliability are assured through a joint effort between the Children’s Hospital Association and participating hospitals. Participating hospitals provide encounter-level data, including demographics, diagnoses, procedures, and measures of resource use. Complex chronic conditions were identified using diagnoses, procedures, measures of resource use, and associated complex chronic conditions.^17^ Race and ethnicity were determined by site-specific practices at each participating PHIS hospital, including self-report by a child’s guardian. We categorized race and ethnicity into 5 groups: Asian, Black, Hispanic, White, and other (including American Indian, multiracial, other race, and missing).

The COI measures neighborhood resources and conditions across more than 72 000 US Census tracts (ie, neighborhoods) from all 50 states and Washington, DC, using data from numerous sources, including the National Center for Education Statistics and the US Department of Education, and is publicly available.^13,18^ The COI includes 29 indicators across 3 domains: (1) educational (eg, third grade math and reading proficiency, high school graduation rates), (2) health and environmental (eg, proximity to grocery stores, proximity to parks and open spaces), and (3) socioeconomic opportunities (eg, unemployment rate, proximity to employment). The COI provides scale scores (range, 1-100) and quintile categorical scores (very low, low, moderate, high, and very high, ) for each domain and a composite for all Census tracts and zip codes in the US. Because the PHIS only includes zip codes, we used the COI at the zip code level. Notably, zip codes were assigned at the encounter level and could change if a patient moved between encounters.

### Outcomes

The primary outcomes were readmissions for DKA within 30 days and within 365 days of an index visit resulting in hospitalization. For children with multiple admissions, each hospitalization was considered an index admission regardless of the number of days since the previous DKA admission. Readmissions were coded as binary (yes or no) variables based on the number of days between the date of discharge and the date of the next admission with a primary diagnosis of DKA. Patients readmitted within 30 days were included among those readmitted within 365 days. Secondary outcomes included the proportion of encounters with codes for AKI (*ICD-9*: 584.5-584.9 and 586; *ICD-10*: N17.0-N17.2, N17.8, N17.9, and N19) or CE (*ICD-9*: 348.5; *ICD-10*: G93.6) at the index visit. Acute kidney injury and CE were selected as secondary outcomes given the commonality of AKI^19,20,21,22^ and the mortality associated with CE.^17,23,24,25^

### Statistical Analysis

Data were analyzed from April 29, 2021, to January 5, 2022. We used descriptive statistics to summarize encounter-level demographics and clinical outcomes overall and by COI category (very low, low, moderate, high, and very high). Patient-level characteristics were defined with respect to the patient’s incident encounter.

We used mixed-effects logistic regression to estimate the association between neighborhood COI and our binary outcomes: (1) 30-day readmission, (2) 365-day readmission, (3) AKI, and (4) CE, with random intercepts for hospital and patient. Models for 30- and 365-day readmissions were adjusted for the following covariates: age, sex, payer, complex chronic condition, intensive care unit admission, concurrent diagnosis of AKI or CE, and discharge year. In addition, we tested the significance of the interaction between race and ethnicity and COI category using a Wald test for each outcome. Nonsignificant interaction terms were excluded from the final model. Results for our regression models are reported as probabilities standardized to the distribution of the covariates in our sample and percentage-point risk differences with 95% CIs using the postestimation margins command in Stata.^26^ To summarize the proportion of variation in our outcomes explained by hospital and patient random effects, we used intraclass correlation coefficients. All analyses were conducted using Stata, version 16 (StataCorp LLC), and 2-sided *P* < .05 was considered statistically significant.

## Results

**Figure 1.**
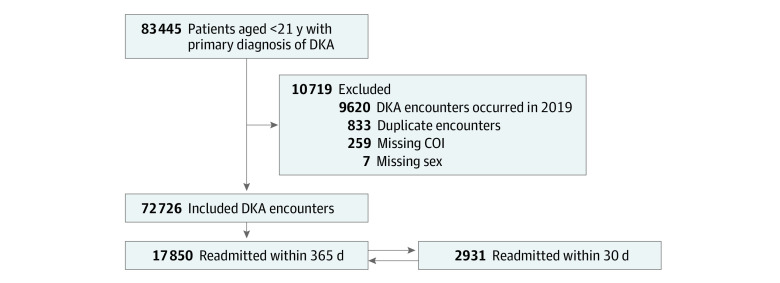
Study Flowchart COI indicates Child Opportunity Index 2.0; DKA, diabetic ketoacidosis. Encounters for which patients were readmitted within 30 days were included for those readmitted within 365 days in the analysis.

A total of 83 445 DKA encounters were identified, of which 72 726 were included in our analysis (median age, 13 [IQR, 9-15] years; 38 924 for girls [53.5%] and 33 802 for boys [46.5%]) (Table 1), representing 46 496 unique patients from 49 hospitals. In terms of race and ethnicity, 600 (0.8%) of the encounters occurred in Asian patients, 16 876 (23.2%) occurred in Black patients, 9969 (13.7%) occurred in Hispanic patients, 40 129 (55.2%) occurred in White patients, and 5152 (7.1%) occurred in patients of other race or ethnicity. Overall, 2931 encounters (4.0%) resulted in a readmission for DKA within 30 days and 17 850 (24.5%) within 365 days of the index encounter (Figure 1).

**Table 1.** Demographic and Encounter Characteristics by COI Category

Characteristic	Encounters by COI category^a^					
	All (N = 72 726)	Very low (n = 17 263)	Low (n = 15 271)	Moderate (n = 14 599)	High (n = 12 940)	Very high (n = 12 653)
COI domain scores, mean (SD)^b^						
Socioeconomic	48 (29)	11 (7)	32 (8)	52 (9)	71 (9)	90 (7)
Health and environment	44 (28)	16 (16)	32 (19)	47 (21)	63 (20)	76 (17)
Educational	47 (29)	15 (12)	33 (15)	48 (16)	66 (15)	86 (10)
Age, y						
Median (IQR)	13 (9-15)	13 (10-16)	13 (10-15)	13 (9-15)	12 (9-15)	12 (9-15)
<6	7578 (10.4)	1344 (7.8)	1493 (9.8)	1528 (10.5)	1588 (12.3)	1625 (12.8)
6-11	21 789 (30.0)	4787 (27.7)	4448 (29.1)	4301 (29.5)	4018 (31.1)	4235 (33.5)
>11	43 359 (59.6)	11 132 (64.5)	9330 (61.1)	8770 (60.1)	7334 (56.7)	6793 (53.7)
Sex						
Girls	38 924 (53.5)	9531 (55.2)	8311 (54.4)	7894 (54.1)	6690 (51.7)	6498 (51.3)
Boys	33 802 (46.5)	7732 (44.8)	6960 (45.6)	6705 (45.9)	6250 (48.3)	6155 (48.6)
Race and ethnicity						
Asian	600 (0.8)	68 (0.4)	83 (0.5)	117 (0.8)	103 (0.8)	229 (1.8)
Black	16 876 (23.2)	8198 (47.5)	3492 (22.9)	2532 (17.3)	1652 (12.8)	1002 (7.9)
Hispanic	9969 (13.7)	3333 (19.3)	2473 (16.2)	1958 (13.4)	1352 (10.4)	853 (6.7)
White	40 129 (55.2)	4511 (26.1)	8135 (53.3)	9014 (61.7)	8943 (69.1)	9526 (75.3)
Other^c^	5152 (7.1)	1153 (6.7)	1088 (7.1)	978 (6.7)	890 (6.9)	1043 (8.2)
Insurance						
Government	38 706 (53.2)	12 757 (73.9)	9478 (62.1)	7644 (52.4)	5334 (41.2)	3493 (27.6)
Private	31 734 (43.6)	4010 (23.2)	5387 (35.3)	6379 (43.7)	7218 (55.8)	8740 (69.1)
Complex chronic condition	3093 (4.3)	864 (5.0)	629 (4.1)	598 (4.1)	533 (4.1)	469 (3.7)
Hospital LOS, median (IQR), d	2 (1-3)	2 (1-3)	2 (1-3)	2 (1-3)	2 (1-3)	2 (1-3)
ICU						
Admission	27 689 (38.1)	6834 (39.6)	6005 (39.3)	5874 (40.2)	4685 (36.2)	4291 (33.9)
LOS, median (IQR), d	1 (1-1)	1 (1-1)	1 (1-1)	1 (1-1)	1 (1-1)	1 (1-1)
Outcomes						
Readmission						
Within 30 d	2931 (4.0)	1047 (6.1)	601 (3.9)	558 (3.8)	445 (3.4)	280 (2.2)
Within 365 d	17 850 (24.5)	5994 (34.7)	4065 (26.6)	3499 (24.0)	2506 (19.4)	1786 (14.1)
AKI	3592 (4.9)	884 (5.1)	764 (5.0)	776 (5.3)	573 (4.4)	595 (4.7)
CE	1029 (1.4)	254 (1.5)	211 (1.4)	217 (1.5)	175 (1.3)	172 (1.3)

Abbreviations: AKI, acute kidney injury; CE, cerebral edema; COI, Child Opportunity Index 2.0; ICU, intensive care unit; LOS, length of stay; PHIS, Pediatric Health Information System.

^a^ Unless otherwise indicated, data are expressed as number (%) of encounters. Percentages have been rounded and may not total 100.

^b^ Scores range from 0 to 100, with higher scores indicating greater opportunity.

^c^ Includes American Indian, multiple races, listed as other when submitted to PHIS by participating hospital, and missing.

The probability of readmission for DKA was associated with COI category (eFigure in the Supplement). Readmission for DKA within 365 days was highest for children living in very low–opportunity neighborhoods (19.2% [95% CI, 17.8%-20.5%]) (Table 2). Comparatively, the probability of readmission for children in very high–opportunity neighborhoods was 5.4 percentage points lower (13.8% [95% CI, 12.6%-15.0%]). The interaction between racial and ethnic groups and COI category was statistically significant (*P* = .04).

**Table 2.** Probability of Readmission at 30 and 365 Days, Acute Kidney Injury, and Cerebral Edema by Race and Ethnicity and COI Category

Variable	Probability of outcome by COI category, % (95% CI)				
	Very low	Low	Moderate	High	Very high
**30-d Readmissions**						
Overall	3.7 (3.3 to 4.1)	3.1 (2.7 to 3.5)	3.2 (2.8 to 3.6)	3.1 (2.7 to 3.5)	2.5 (2.1 to 2.9)
Race and ethnicity					
Asian	2.7 (−1.4 to 6.8)	2.3 (−1.0 to 5.6)	4.1 (0.2 to 8.0)	1.3 (1.2 to 3.8)	0.7 (−0.7 to 2.1)
Black	5.1 (4.3 to 5.9)	3.9 (3.1 to 4.7)	3.9 (3.1 to 4.7)	4.0 (3.0 to 5.0)	4.8 (3.4 to 6.2)
Hispanic	3.3 (2.5 to 4.1)	2.6 (2.0 to 3.2)	2.5 (1.7 to 3.3)	3.2 (2.2 to 4.2)	2.2 (1.2 to 3.2)
White	3.2 (2.6 to 3.8)	2.8 (2.4 to 3.2)	3.0 (2.6 to 3.4)	2.8 (2.4 to 3.2)	2.1 (1.7 to 2.5)
Other	2.0 (1.2 to 2.8)	2.9 (1.9 to 3.9)	2.5 (1.5 to 3.5)	1.7 (0.7 to 2.7)	1.7 (0.9 to 2.5)
**365-d Readmissions**						
Overall	19.2 (17.8 to 20.5)	17.2 (16.0 to 18.4)	16.2 (15.0 to 17.4)	15.3 (14.1 to 16.8)	13.8 (12.6 to 15.0)
Race and ethnicity					
Asian	14.5 (5.9 to 23.1)	11.4 (5.9 to 16.9)	9.0 (3.1 to 14.9)	4.5 (0.2 to 8.8)	4.8 (1.5 to 8.1)
Black	24.9 (23.1 to 26.7)	22.8 (20.8 to 24.8)	21.1 (18.9 to 23.3)	21.2 (18.8 to 23.6)	20.4 (17.7 to 23.1)
Hispanic	17.1 (15.4 to 18.9)	16.6 (14.8 to 18.5)	15.5 (13.6 to 17.4)	15.9 (13.7 to 18.2)	13.9 (11.4 to 16.5)
White	17.4 (15.8 to 19.0)	14.9 (13.5 to 16.3)	14.6 (13.4 to 15.8)	13.0 (11.8 to 14.2)	11.2 (10.0 to 12.4)
Other	16.1 (13.7 to 18.5)	16.6 (14.2 to 19.0)	12.7 (10.3 to 15.1)	11.0 (8.6 to 13.4)	9.9 (7.7 to 12.2)
**Acute kidney injury**						
Overall	5.6 (4.2 to 7.0)	5.3 (3.9 to 6.7)	5.4 (4.0 to 6.8)	5.0 (3.6 to 6.4)	4.8 (3.4 to 6.2)
Race and ethnicity					
Asian	6.7 (0.4 to 13.3)	2.8 (−0.7 to 6.3)	5.1 (0.4 to 9.8)	4.8 (0.7 to 8.9)	5.8 (2.7 to 8.9)
Black	7.2 (5.4 to 9.0)	7.1 (5.1 to 9.1)	6.5 (4.7 to 8.3)	6.9 (4.7 to 9.1)	5.7 (3.7 to 7.7)
Hispanic	4.1 (2.7 to 5.5)	4.2 (2.8 to 5.6)	4.6 (3.0 to 6.2)	4.7 (3.1 to 6.3)	3.8 (2.2 to 5.4)
White	5.5 (3.9 to 7.1)	4.9 (3.5 to 6.3)	4.8 (3.4 to 6.2)	4.4 (3.2 to 5.6)	4.5 (3.3 to 5.7)
Other	5.4 (3.4 to 7.4)	4.7 (2.9 to 6.5)	6.4 (4.2 to 8.6)	5.1 (3.1 to 7.1)	4.3 (2.7 to 5.9)
**Cerebral edema**						
Overall	1.9 (1.5 to 2.3)	1.6 (1.2 to 2.0)	1.7 (1.3 to 2.1)	1.5 (1.1 to 1.9)	1.4 (1.0 to 1.8)
Race and ethnicity					
Asian	1.7 (−1.6 to 5.0)	0.9 (−0.9 to 2.7)	0.9 (−0.9 to 2.7)	1.0 (−1.0 to 3.0)	0.5 (−0.5 to 1.5)
Black	2.1 (1.5 to 2.7)	2.0 (1.2 to 2.8)	1.4 (0.8 to 2.4)	1.8 (1.0 to 2.6)	1.5 (0.5 to 2.5)
Hispanic	1.6 (1.0 to 2.2)	1.3 (7.0 to 1.9)	1.4 (0.8 to 2.0)	1.4 (0.8 to 2.0)	1.1 (0.3 to 1.9)
White	1.8 (1.2 to 2.4)	1.6 (1.2 to 2.0)	1.8 (1.2 to 2.4)	1.4 (1.0 to 1.8)	1.5 (1.1 to 1.9)
Other	1.8 (1.0 to 2.6)	1.8 (0.8 to 2.85)	1.5 (0.7 to 2.3)	1.6 (0.8 to 2.4)	1.3 (0.5 to 2.1)

Abbreviation: COI, Child Opportunity Index 2.0.

**Figure 2.**
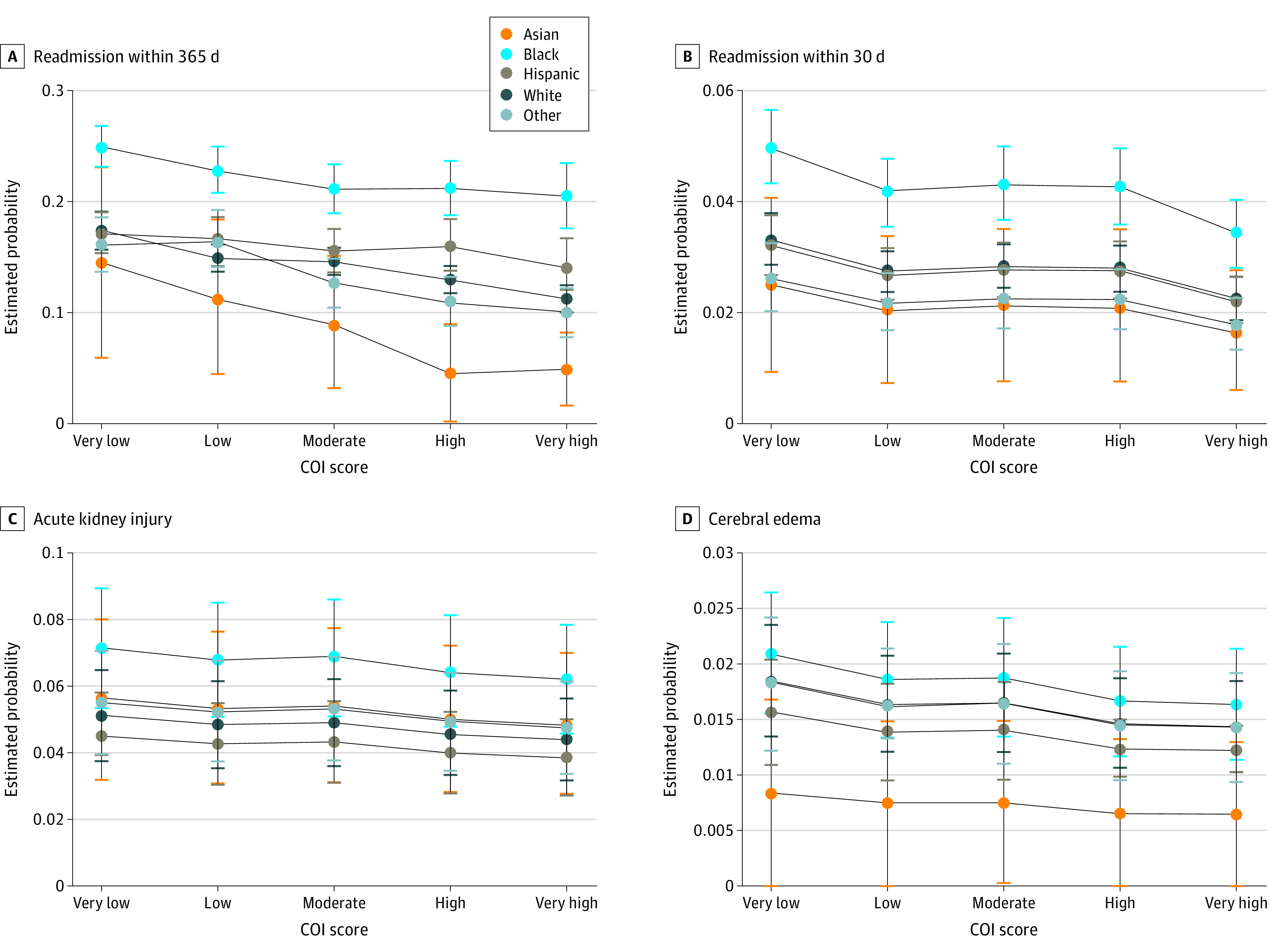
Probabilities of the Main Outcomes of Readmission and Diabetes-Related Complications by Race and Ethnicity and Overall Neighborhood Child Opportunity Child opportunity index 2.0 (COI) categorical scores at or below the 20th percentile were categorized as very low; greater than 20th to at or below the 40th percentile, as low; greater than 40th to at or below the 60th percentile, as moderate; greater than 60th to at or below the 80th percentile, as high; and greater than 80th percentile, as very high. Whiskers represent 95% CIs.

At all levels of overall COI, Black children had a greater probability of readmission compared with Hispanic children and White children (Figure 2 and Table 2). Across racial groups, the probability of readmission within 365 days of an index encounter was significantly higher among Black children with an overall very low COI category compared with White children (risk difference, 7.5 [95% CI, 5.9-9.1] percentage points) and Hispanic children (risk difference, 7.8 [95% CI, 6.0-9.6] percentage points) at the same level of COI (Table 2). Similar racial differences were seen for children with an overall very high COI category (risk difference for Black compared with White children, 9.3 [95% CI, 6.6-11.9] percentage points; risk difference for Black compared with Hispanic children, 6.5 [95% CI, 4.4-8.6] percentage points) (Table 2). Within racial groups, Hispanic (risk difference, 3.2 [95% CI, 0.4-5.9] percentage points), Black (risk difference, 4.5 [95% CI, 1.7-7.2] percentage points), and White (risk difference, 6.2 [95% CI, 4.9-7.6 percentage points]) children with an overall very low COI category had significantly greater probability of readmission within 365 days compared with those with an overall very high COI category. Patient random effects explained approximately 40% of the variation in 365-day readmission (intraclass correlation coefficient, 0.40 [95% CI, 0.38-0.41]). Only minor variation in our outcome was explained by hospital effects (intraclass correlation coefficient, 0.02 [95% CI, 0.01-0.04]).

**Figure 3.**
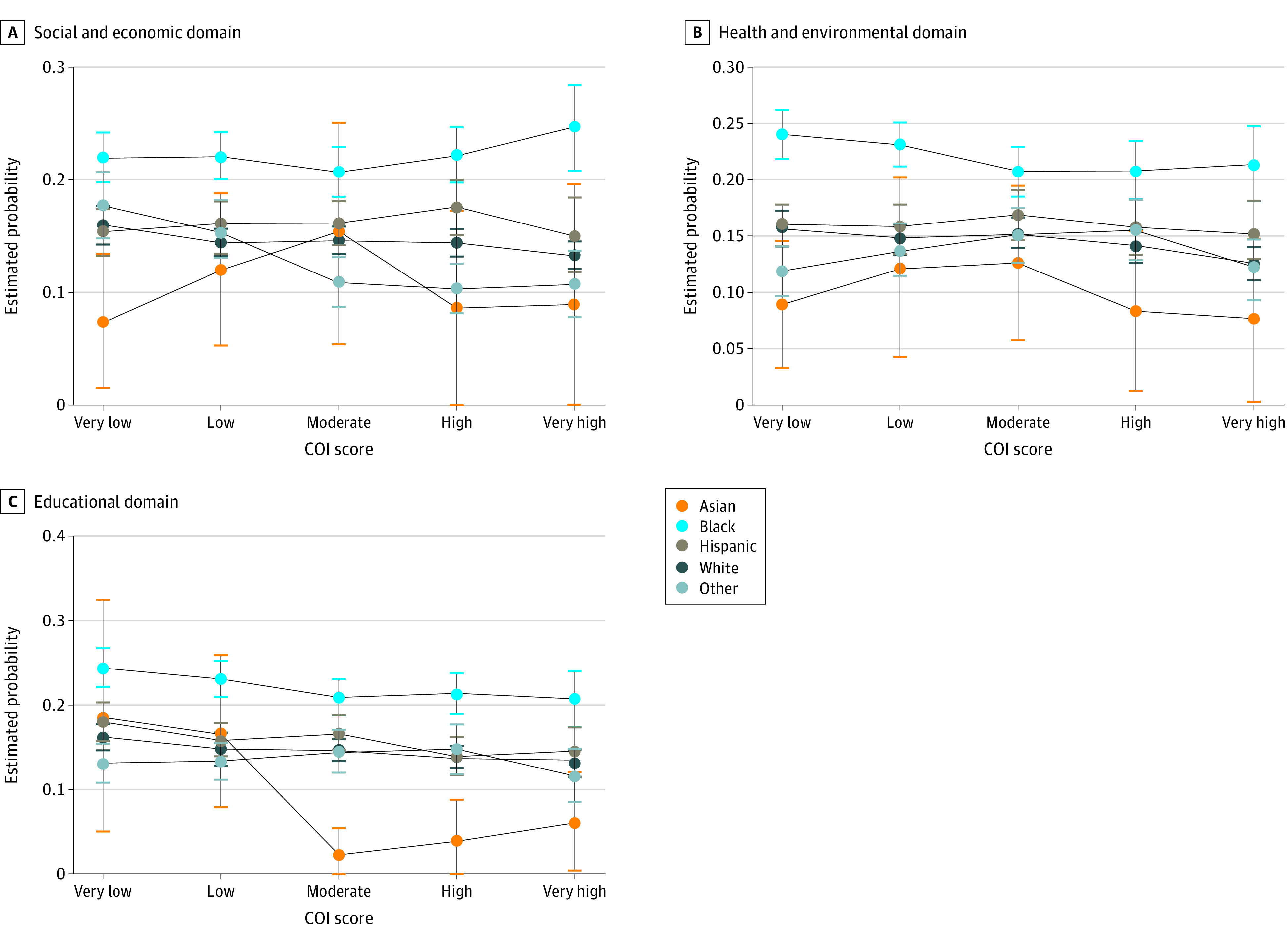
Probability of Readmissions Within 365 Days by Race and Ethnicity and Neighborhood Child Opportunity Domains Child opportunity index 2.0 (COI) categorical scores at or below the 20th percentile were categorized as very low; greater than 20th to at or below the 40th percentile, as low; greater than 40th to at or below the 60th percentile, as moderate; greater than 60th to at or below the 80th percentile, as high; and greater than 80th percentile, as very high. Whiskers represent 95% CIs.

For each of the 3 COI domains, Black children had a greater probability of 365-day readmission at all COI categories compared with Hispanic children and White children (Figure 3). Among children with very low socioeconomic COI domains, the probability of readmission within 365 days was significantly higher compared with that of White children (risk difference, 6.2 [95% CI, 4.0-8.4] percentage points) and Hispanic children (risk difference, 6.7 [95% CI, 4.1-9.3] percentage points) with very low socioeconomic COI domains (eTable in the Supplement). The interaction between racial and ethnic group and COI domain was statistically significant for the socioeconomic domain (Wald χ^2^ = 30.22; *P* = .02). Similar results were seen for the health and environment and educational domains.

Category of COI was not associated with AKI; the probability of AKI was 5.6% among children with an overall very low COI category compared with 4.8% among those with an overall very high COI category (risk difference, 0.8 [95% CI, 0-1.3] percentage points); frequency was 4.9% among all categories. Race and ethnicity did not modify the association between COI category and AKI (Wald χ^2^ = 13.4; *P* = .65). However, race and ethnicity were independently associated with AKI (Figure 2 and Table 2). The probability of AKI was 6.8% among Black children compared with 4.8% among White children (risk difference, 2.0 [95% CI, 1.3-2.6] percentage points) and 4.2% among Hispanic children (risk difference, 2.5 [95% CI, 1.7-3.3] percentage points). Neither COI nor race and ethnicity were significantly associated with CE, and there was no evidence of effect modification (Wald χ^2^ = 6.82; *P* = .96) (Figure 2 and Table 2).

## Discussion

Our findings suggest that Black children experience disparities in T1D care despite having similar measures of neighborhood opportunity when compared with other racial groups. Using data from 49 US children’s hospitals, we observed that Black children had greater probability of readmission for DKA compared with Hispanic children and White children at all COI categories. Within racial and ethnic groups, children with an overall very low COI category had significantly greater probability of readmission compared with those with an overall very high COI category. Our results have implications for health systems seeking to reduce disparities.

Hospital readmissions are commonly used as a quality indicator, in part because they may be avoidable with appropriate care during the index hospitalization and sufficient ambulatory care after discharge. This concept is the motivation for financial penalties associated with pay-for-performance strategies, such as the Centers for Medicare & Medicaid Services Hospital Readmission Reduction Program^27^ for adults and the Potentially Preventable Readmission measure used for pediatric readmissions.^28,29^ Although the use of readmissions as a quality indicator is controversial,^30,31^ the burden to families and health systems is undeniable. It has been estimated that 30-day pediatric readmissions account for $678 million dollars of all annual health care costs.^32^ For children with T1D, the median charge for a DKA readmission is greater than $12 000,^1^ and pediatric intensive care unit charges are even higher.^33^ Children who are readmitted also experience length of stays more than double those of patients who are not readmitted,^34^ making the burden on families even greater as they spend more time away from work and incur personal expenses. Further, the burden of readmission may disproportionately affect families who identify as Black and/or have public insurance, both of which are associated with increased readmission rates.^35,36,37^

Investigation has shown that there is widespread variation in readmissions for DKA across US children’s hospitals.^1^ A 2017 study^38^ showed that 365-day readmissions were significantly higher for Black children with T1D (21.7%) compared with children of other races (13.4%). In another study using PHIS data from 2004 to 2012,^7^ 365-day readmissions for DKA occurred in 28% of children, and factors associated with readmission included public insurance and Black race. Both findings are consistent with our results. Further, the authors of one of these studies^7^ showed that Black race was associated with higher odds of 365-day readmission in 59% of hospitals, suggesting variation in DKA admission frequency across hospitals. It is unclear what drives this variation, although a number of factors likely contribute, including implicit biases^39,40^ and possibly neighborhood factors. More recently, Maxwell et al^9^ conducted a retrospective population-based cohort study using data from Cincinnati Children’s Hospital from 2011 to 2017 and examined whether Census tract poverty, race, and insurance status were associated with hospitalization for DKA in children. The authors conducted a subanalysis among children who were readmitted and found that Black race, public insurance status, and greater Census tract poverty were significantly associated with readmission.^9^ However, this study was limited in that the number of readmissions was low, with only 42 children (10%) being readmitted during the study period.^9^

Several studies^14,15,16^ have begun to explore the association among neighborhood opportunity, acute care use, and hospitalizations. One study found that residing in an area in the very low COI category was associated with significantly greater odds of having multiple acute care visits for conditions amenable to outpatient care compared with children who live in areas in the very high COI category.^14^ A similar study found that the hospitalization rate for asthma was significantly higher at 9.1 per 1000 children living in areas in the very low COI category compared with 1.8 per 1000 for those living in areas in the very high COI category.^15^ Area deprivation, a measure of neighborhood opportunity derived from American Community Survey data, has recently been associated with greater odds of hospital admission for recurrent DKA.^41^ However, that study was limited to a single state, which reduces generalizability. Only 1 previous study^16^ has evaluated use of diabetes resources in association with COI categories. Krager et al^16^ found that the hospitalization rate for children with diabetes and associated complications was significantly higher at 2.8 per 1000 children for those living in areas with a very low COI category compared with 1.5 per 1000 children in areas with a very high COI category.^16^ In the present study, we expand on these findings, using data from the largest children’s hospitals merged with COI data.

Our results provide an equity-focused evaluation of disparities as they are associated with diabetes-related outcomes. The COI includes measures such as reading and math proficiency, adult educational attainment, and access to healthy food, all of which are particularly important for children living with T1D because they must calculate insulin dosing, manage diabetes technology (eg, insulin pumps), and find healthy food choices. The importance is further supported by previous work showing that the risk of DKA and glycemic excursions are associated with lower parental educational level^10,11,42^ and unhealthy dietary intake choices.^43,44,45,46^ In addition, household food insecurity has been associated with higher hemoglobin A_1c_ values and hospitalization rates in children with T1D,^47^ which is notable because children with lower COI categories have more limited access to healthy food choices compared with those with higher COI categories.^13,48,49^ Less frequent use of diabetes technology, particularly continuous glucose monitoring devices, has also been associated with worse health outcomes in children with T1D, including higher rates of DKA^50^ and time spent in hyperglycemia.^51^ In a recent study of 1500 children, Lai et al^52^ showed that 54% of White children with T1D started continuous glucose monitoring in the outpatient setting compared with 31% of Black and 33% of Hispanic children. It is unclear why individuals from racial and ethnic minority groups less frequently start continuous glucose monitoring, but it is notable that this disparity persisted even after adjusting for insurance status,^52^ suggesting that lack of coverage may not be a driving factor. The COI includes many socioeconomic measures; however, there are other unmeasured factors that may also contribute to disparities. For instance, lack of reliable transportation and concern for missing work, which disproportionally affect individuals from racial and ethnic minority groups and those with lower socioeconomic status,^53^ may influence a family’s ability to attend clinic visits.^54,55,56^ Previous studies have shown that children with at least 2 missed clinic visits have significantly higher hemoglobin A_1c_ levels compared with children with 1 or no missed visits,^57,58^ and missing endocrinology visits has been shown to be associated with higher odds of hospitalization for DKA.^59^ With these factors in mind, it is not surprising that children with lower COI levels, and particularly Black children, are at risk for DKA readmissions.

We also found that diabetes-related AKI was associated with race and ethnicity but not COI category. Although studies among adults with T1D have demonstrated racial differences in AKI^60,61,62^ and recent work among children has highlighted the frequency of AKI in children with DKA,^19,20,21,22^ no studies to our knowledge have explicitly studied the association between race and ethnicity and diabetes-related AKI risk in the pediatric population. Thus, our finding that race and ethnicity constituted a significant factor associated with AKI across all levels of COI is novel, and future research should focus on racial and ethnic disparities in AKI among children with DKA. In terms of CE risk, we found no association between COI category or race and ethnicity and risk of CE. To our knowledge, no studies have explicitly examined the association between race and ethnicity and CE risk in children with DKA, likely because CE is a rare event, and large sample sizes would be needed to detect any differences.

### Limitations

Our study has several limitations. First, we could not account for readmissions to non-PHIS hospitals, which precluded our ability to evaluate patient-level data. Second, factors not included in the COI may contribute to readmissions. For instance, exposure to adverse childhood experiences has been shown to have an association with hospitalization rates.^63^ It is also possible that other patient or clinician factors contribute to readmissions. We were unable to account for the presence of coexisting mental health conditions, which have been shown to be associated with readmissions for DKA in children.^7,64^ Implicit bias by clinicians may further influence treatment decisions, including hospitalization.^39,65^ Third, we were unable to reliably account for use of diabetes technology, which has been associated with lower rates of DKA.^50,66,67^ For example, the prevalence of the *ICD-10* code for insulin pump use (Z96.41) in PHIS was only 3% and varied significantly by hospital from less than 1% to 18%. Documentation of insulin pump use has also been an issue in other administrative data sets.^67^ Fourth, the COI uses Census tract data, which may not align with how individuals perceive their neighborhood boundaries. This factor is important to recognize as interventions aimed at addressing health disparities are designed and implemented. Fifth, the frequency of AKI in our cohort was found to be 4.9%, which is lower in comparison with recent reports.^20,21,22^ This lower rate is likely due to ascertainment of AKI by *ICD-9* and *ICD-10* coding because we did not have access to laboratory values, whereas other studies were able to use creatinine data to define AKI.

## Conclusions

Our study revealed significant disparities in DKA health outcomes associated with neighborhood opportunity. Readmissions were higher in patients with low COI categories, adding strain to already disadvantaged populations. The results of our study may be useful both for clinicians and health care systems as they seek ways to reduce health disparities and advocate for patients and families as well as for policy makers and community leaders who seek to enact change on a population level.

## Notes

Supplement. **eTable.** Estimated Probability of Readmission for Each COI Domain by Race and Ethnicity **eFigure.** Projective Margins for DKA Readmission at 365 Days by Overall Neighborhood Child Opportunity, Adjusted for Race and Ethnicity
