# Supplementary material for: Association of Neighborhood Resources and Race and Ethnicity With Readmissions for Diabetic Ketoacidosis at US Children’s Hospitals
Source: JAMA Netw Open. 2022 May 5;5(5):e2210456. doi: 10.1001/jamanetworkopen.2022.10456 (PMC9073568; doi:10.1001/jamanetworkopen.2022.10456)
Supplement: Supplement. — eTable. Estimated Probability of Readmission for Each COI Domain by Race and Ethnicity eFigure. Projective Margins for DKA Readmission at 365 Days by Overall Neighborhood Child Opportunity, Adjusted for Race and Ethnicity [file jamanetwopen-e2210456-s001.pdf]

Supplementary Online Content

Bergmann KR, Nickel A, Hall M, et al. Association of neighborhood resources and race and ethnicity with readmissions for diabetic ketoacidosis at US children's hospitals. *JAMA Netw Open*. 2022;5(5):e2210456. doi:10.1001/jamanetworkopen.2022.10456

eTable. Estimated Probability of Readmission for Each COI Domain by Race and Ethnicity
eFigure. Projective Margins for DKA Readmission at 365 Days by Overall Neighborhood Child Opportunity, Adjusted for Race and Ethnicity

This supplementary material has been provided by the authors to give readers additional information about their work.

© 2022 Bergmann KR et al. *JAMA Network Open*.

eTable. Estimated Probability of Readmission for Each COI Domain by Race and Ethnicity										
	**Very Low**		**Low**		**Moderate**		**High**		**Very High**	
**COI Domain**	**Readmit (%)**	**SE**	**Readmit (%)**	**SE**	**Readmit (%)**	**SE**	**Readmit (%)**	**SE**	**Readmit (%)**	**SE**
Social & Economic										
Overall	17.6	0.7	16.9	0.6	16.3	0.6	16.8	0.7	16.5	0.8
NH-black	22.1	1.1	22.1	1.1	20.8	1.1	22.2	1.4	24.7	1.9
NH-white	15.9	0.8	14.6	0.7	14.6	0.7	14.6	0.7	13.4	0.8
Hispanic	15.4	1.1	16.2	1.0	16.1	1.0	17.6	1.3	15.1	1.7
Asian	7.5	3.0	11.9	3.5	15.3	.5.0	8.7	4.6	9.0	5.5
Other	17.8	1.6	15.4	1.3	11.0	1.3	10.5	1.2	10.8	1.5
Health & Environment										
Overall	17.4	0.7	16.8	0.6	16.7	0.6	15.9	0.7	15.0	0.8
NH-black	24.0	1.1	23.2	1.0	20.7	1.1	20.8	1.3	21.4	1.8
NH-white	15.7	0.8	14.7	0.7	15.3	0.7	14.0	0.7	12.6	0.7
Hispanic	16.1	1.0	15.9	1.0	16.9	1.1	15.8	1.2	15.2	1.5
Asian	8.9	2.9	12.1	4.0	12.6	3.5	8.3	3.6	7.7	3.7
Other	11.9	1.1	13.7	1.1	15.1	1.3	15.5	1.3	12.3	1.5
Education										
Overall	18.2	0.7	16.8	0.6	16.3	0.6	15.7	0.7	15.0	0.8
NH-black	24.4	1.2	23.1	1.1	20.9	1.1	21.3	1.2	20.7	1.7
NH-white	16.3	0.8	14.7	0.7	14.7	0.7	13.9	0.7	13.2	0.8
Hispanic	18.1	1.2	15.8	1.0	16.7	1.0	13.9	1.1	14.4	1.5
Asian	18.7	7.0	16.8	4.6	2.2	1.6	4.2	2.3	6.1	2.9
Other	13.1	1.2	13.4	1.2	14.5	1.3	14.8	1.5	11.4	1.6

COI – Child Opportunity Index; NH – Non-Hispanic; SE – Standard Error.

© 2022 Bergmann KR et al. *JAMA Network Open*.

eFigure. Projective Margins for DKA Readmission at 365 Days by Overall Neighborhood Child Opportunity, Adjusted for Race and Ethnicity

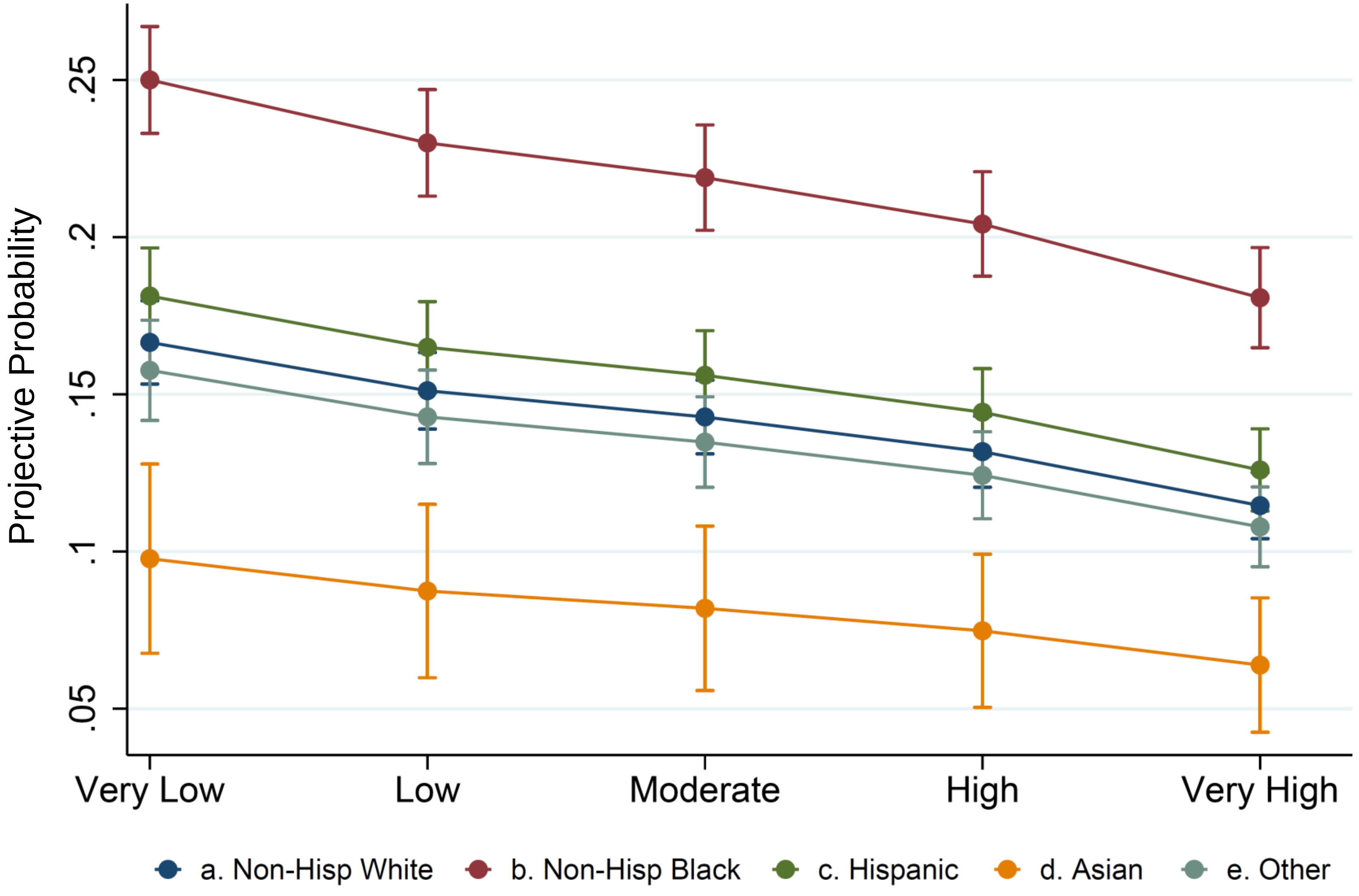

© 2022 Bergmann KR et al. *JAMA Network Open*.
